# Daycare shooting in Thailand: Time to address firearm violence as a global health issue

**DOI:** 10.1002/puh2.93

**Published:** 2023-06-01

**Authors:** Yasmine Syifa Nabila Budi, Fabiola Cathleen, Manatee Jitanan, Oraphan Butkatunyoo, Adriana Viola Miranda

**Affiliations:** ^1^ Faculty of Medicine Universitas Indonesia Jakarta Indonesia; ^2^ Department of Physical Education, Faculty of Education Kasetsart University Bangkok Thailand; ^3^ Department of Education, Faculty of Education Kasetsart University Bangkok Thailand; ^4^ Global Health Focus Asia Bandung Indonesia

**Keywords:** firearm safety, firearm violence, gun violence, Thailand

## Abstract

On 6 October 2022, a daycare shooting incident happened in Thailand. It is the deadliest mass shooting in Southeast Asian (SEA) countries and one of the many incidents contributing to the global epidemic of firearm‐related preventable violence. Although firearm violence is commonly associated with the United States, it is also a problem worldwide, causing long‐lasting health issues and huge economic burden. This is due to the root causes of firearm violence transcending national boundaries, including socioeconomic landscape, political dynamics, and globalization. Considering these and the major public health impacts of firearm violence, firearm violence should be prioritized as a global health issue. We identified public health challenges related to global firearm violence management: inadequate regulation, lack of official firearm surveillance, and inadequate access to mental health care. In Thailand, there is a gap in gun ownership policies for the civilian and government personnel and a relatively less strict background check compared to other SEA countries. It is crucial for the governments to strengthen their firearm regulations and invest in appropriate public health interventions, including improving access to mental health services and conducting firearm‐related surveillance and culturally sensitive awareness programs. Various public health stakeholders, including healthcare workers and local communities, should be engaged to effectively mitigate future firearm violence.

## INTRODUCTION

On 6 October 2022, a 34‐year‐old former police officer stormed a child development center in Nong Bua Lamphu province at about 12:30 local time. He was understood to appear under the influence of drugs. Armed with a knife, a shotgun, and a pistol, he killed at least 38 people, including 24 children, and injured 10 others. To date, it is the deadliest mass shooting ever to happen in the Southeast Asian (SEA) region [1]. Although this may seem like an isolated incident, in general, firearm violence is a growing issue in Thailand. According to the Institute of Health Metrics and Evaluation (IHME) at the University of Washington's 2019 Global Burden of Disease database, firearm homicide in Thailand is the second highest in Southeast Asia [2]. This may be related to the high firearm ownership in Thailand: The Switzerland‐based Small Arms Survey (SAS) in 2018 showed that Thailand's firearm ownership ranks first in the SEA region, with more than 10.3 million civilian‐owned firearms or around 15 guns per 100 people. Only 6.2 million are legally registered [3]. Thailand's government has been battling armed insurgency and small‐scale firearm violence as firearm‐control efforts have been challenged by significant smuggling, promoting its availability and affordability [1].

Unfortunately, this seemingly escalating firearms violence is not limited to Thailand but on a global scale. Firearm violence is increasingly prevalent worldwide: An epidemic of firearm‐related preventable violence took a death toll of an estimated 217,000 people worldwide in 2019, 71% of which were due to physical violence by firearm. In particular, the incidence of physical violence by firearm is increasing at an unprecedented rate: By 2019, it has increased by 37.5% compared to 1990 [2]. The consequences of firearm violence go beyond the loss of lives, as it also leads to persisting mental and physical health problems and other social repercussions [2, 4]. For instance, in the United States (US), firearm violence costs more than 150 billion US dollars annually [4]. In Southeast Asia, firearm violence led to disability‐adjusted life years (DALYs) of over 780,000. This is higher than the DALYs due to more commonly discussed health issues in the region, such as fire incidents and measles [2]. This commentary aims to illustrate the challenges and possible solutions to firearm violence as a global health issue.

## FIREARM VIOLENCE AS A GLOBAL HEALTH ISSUE

It is common to compartmentalize firearm violence as a public health issue for the US. In 2022 alone, there have been 609 mass shootings reported in the US [5]. The US is the only country where firearm violence is among the top 5 leading causes of mortality in children, causing 5.6 deaths per 100,000 children [4]. However, firearm violence is now increasingly affecting other countries as it becomes directly influenced by the dynamic between high‐income countries (HICs) and low‐ and middle‐income countries (LMICs). This includes the dynamic in economic trading, political climate, and globalization in general [6]. For instance, HICs can provide extensive supply chains and trades of arms to LMICs on a global scale. This noticeably surged during the COVID‐19 pandemic, with firearms worth more than 90 million US dollars sent to Asia [7]. The political landscape in HICs has also impacted the policymaking process of firearm regulations in LMICs. As a part of the global war on drugs first spearheaded by HICs, drug‐related firearm violence by the governments continues to escalate, particularly due to the increasing drug consumption and drug trafficking cases [8]. Moreover, globalization through various forms of media also has shared the “gun culture” from HICs, opening a wider demand and tightening the competition within firearms markets, resulting in the rise of both lethality and accessibility of firearms [6].

As research on this problem grows, firearm violence has similarities to infectious diseases, in the sense that it is possible to impact anyone from HICs and LMICs when exposed to certain individual and environmental risk factors, such as age, gender, personality, socioeconomic status, educational level, and social networks [6]. Hence, addressing firearm violence requires more than traditional public health interventions: As the root transcends boundaries over countries, the issue should be put on a global scale. This fits the definition of global health, which “emphasizes transnational health issues, determinants, and solutions” [9]. By recognizing firearm violence as a global health issue, measures can be strengthened through restructuring multinational interventions.

## CHALLENGES IN FIREARM VIOLENCE MANAGEMENT: GLOBAL LESSONS

The public health preparedness for firearm violence varies between countries. In Thailand, on paper, the possession of assault weapons is banned for citizens. Civilians with strong reasons to acquire firearms, for example, for self‐defense, must pay a tax of up to 40% to buy a firearm legally. The number of guns and ammunition that can be sold or owned is also limited. However, the rules are a bit different between the rules for civilians and military or government personnel, leaving a big gap and accountability for the civilian population and a very big power relation to the military and the government personnel. Military personnel were allowed to buy personal firearms tax‐free through firearm welfare programs, which were scrapped after this latest incident [10]. In addition, Thai firearm owners, both government officials and civilians, are not required to renew their license during the lifetime of the firearm. The background check for license applicants includes personal conduct, living conditions, income, and criminal records and has been reported as less restrictive for government officials [10, 11].

These policies are less strict compared to other countries in the SEA region. For example, in Singapore and Indonesia, firearm license renewal is required every 2 years. Singapore also employs mental health and medical checkup during the application process. The two countries require applicants to undergo theoretical and practical training courses [12, 13]. At the same time, the SEA region faces some similar public health challenges regarding firearm violence. First, the public health reporting of firearm‐related deaths is uncommon, although several global surveys also include data from this region [14]. This is concerning as surveillance allows insight into the causes and risk factors associated with firearm violence, their impact on the community, and the preventive measures that can be used to control the issue. In addition, access to mental health care remains an issue among these countries, despite these problems being strongly associated with firearm violence. It is known that the perpetrator in the Nong Bua Lamphu attack experienced mood swings and drug issues [1], which can be treated with adequate access to mental health care.

## RECOMMENDATIONS

**FIGURE 1:**
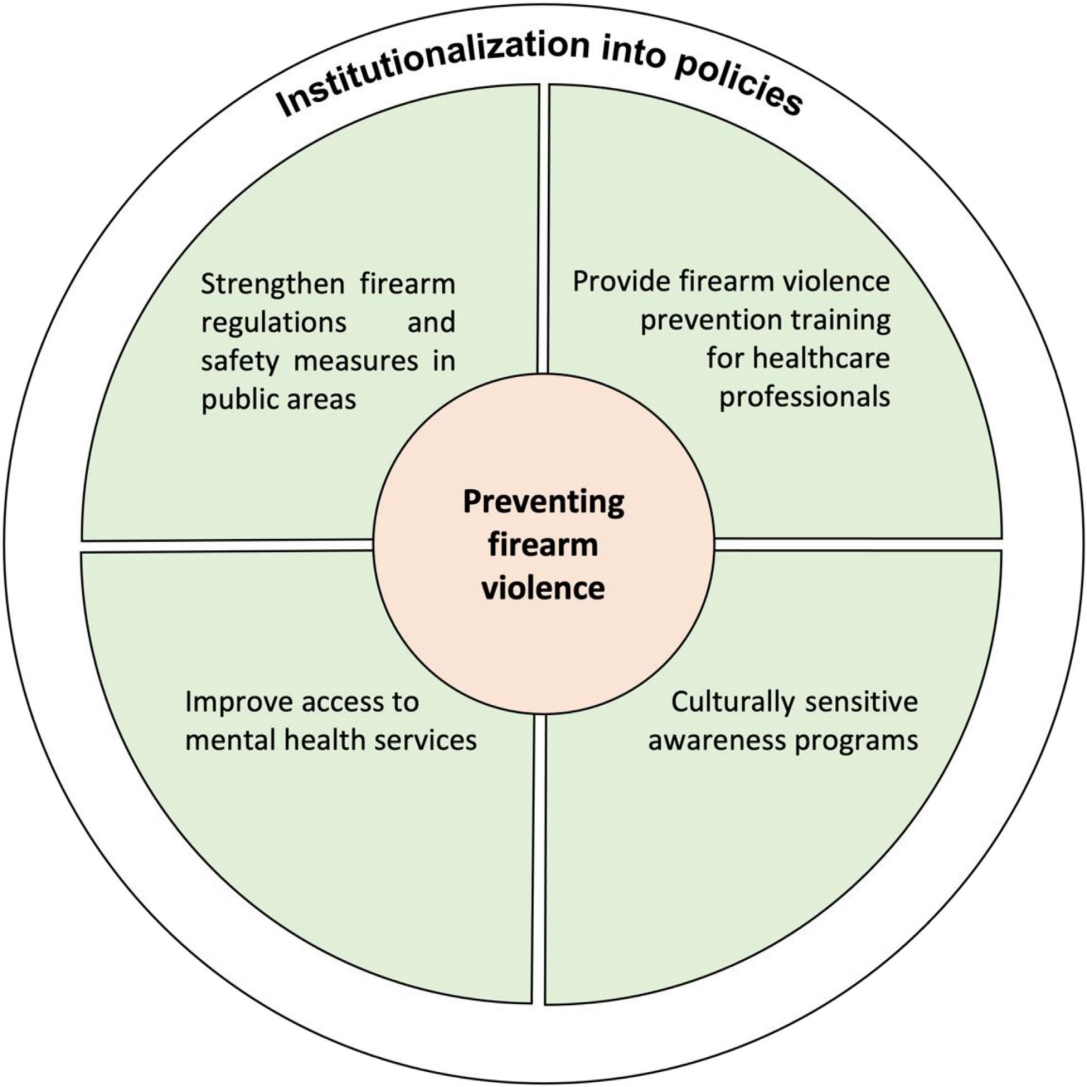
Recommendations to prevent firearm violence.

To effectively control firearm violence, in general, we recommend that governments should recognize the importance of the public health lens in firearm violence management. It is crucial to strengthen firearm control, firearm possession requirements, and law enforcement. Access to mental health services should be prioritized, with additional firearm violence prevention training given to healthcare professionals. Specific programs that protect vulnerable people from firearm violence, especially in places such as child development centers, schools, hospitals, and public places, should be created. This can include conducting awareness programs and strengthening safety measures in these places. Local communities should be involved to curb firearm violence with culturally sensitive approaches. These interventions should be institutionalized into policies and practices, with adequate civilian oversight to improve accountability (Figure 1).

To strengthen national firearm control laws, the remaining loopholes must be addressed. In Thailand, the government can strengthen firearm control by requiring firearm owners to renew/requalify their licenses every few years. Thailand should also employ more robust background checks with public health in mind, by conducting medical and mental health examinations. Mandated training courses should be considered.

In addition, it is time to recognize the value of public health interventions to address this issue. First, firearm‐related surveillance should be of priority, as the data will help in identifying factors related to firearm violence. Second, national governments should collaborate with their health agencies and professional organizations to create clear healthcare and prevention guidelines. Health practitioners should receive adequate training and time to counsel high‐risk patients. Access to mental health services should be improved, with specific focus to high‐risk government officials and civilians that possess firearms.

To incorporate these changes, it may be beneficial to study the public health interventions in the US. Due to the high prevalence of firearm violence in the country, it has extensive firearm‐related surveillance and mental healthcare practices. Prioritization of firearm‐related surveillance can be started by providing adequate and specific funding mechanisms for the projects, as has been done by the Centers for Disease Control and Prevention [15]. Firearm violence–related healthcare guidelines can be developed by contextualizing the guidelines produced in the US, such as the guidelines by the American Academy of Family Physicians (AAFP) and the American Psychological Association. These guidelines state that healthcare professionals can conduct violence risk assessments, provide counseling for high‐risk patients, and educate the general public [16, 17]. It is, however, important to note that physicians have voiced concerns for their safety and liability to effectively follow this guideline. Four main concerns from physicians regarding firearm safety education have been reported: concern about potential liability; physicians being ill‐prepared or uncomfortable discussing firearm safety with their patients; worry about patient cooperativeness; and limited time for effective counseling sessions on firearm safety. These concerns are related to inadequate training on firearm safety counseling medical institutions and the lack of promotion by the government regarding their firearm regulation. Hence, to address these concerns, several training materials have been developed, for example, by the American Academy of Pediatrics (AAP) [15]. These materials can also be contextualized by other countries to match their local situation.

Lastly, community involvement should be prioritized. Effective localization and context‐specific engagement can only be done in collaboration with local stakeholders. Recognizing and implementing a sufficient public health prevention campaign might help the cause and lead to better firearm control and regulation. Adequate civilian oversight will also improve accountability.

## CONCLUSION

The Thailand shooting incident should be a wake‐up call to global governments and the public health community to start addressing firearm violence as a global health issue. Further actions need to be implemented by the government and the public health officials as the leading actors in maintaining a safe community and mitigating the elicit use of guns in the future. Thailand and other countries should strengthen their firearm control policies, involve public health lens and local communities in managing firearm violence, and institutionalize these strategies while ensuring accountability. Implementing these recommendations will mitigate global firearm violence and effectively promote firearm safety.

## AUTHOR CONTRIBUTIONS

*Conceptualization; data curation; formal analysis; writing—original draft; writing—review and editing*: Yasmine Syifa Nabila Budi and Fabiola Cathleen. *Formal analysis; writing—review and editing*: Manatee Jitanan and Oraphan Butkatunyoo. *Conceptualization; supervision; formal analysis; writing—review and editing*: Adriana Viola Miranda.

## CONFLICT OF INTEREST STATEMENT

AVM is an editorial board member of the journal. She was excluded and blinded from all stages of the peer review of this manuscript.
